# Chronic Obstructive Pulmonary Disease Care Preparedness Among Nursing Interns in Egypt: A Cross-Sectional Comparative Study

**DOI:** 10.3390/healthcare14183096

**Published:** 2026-09-20

**Authors:** Yousef S. Aldabayan

**Affiliations:** Department of Respiratory Care, College of Applied Medical Sciences, King Faisal University, Al-Ahsa 31982, Saudi Arabia; yaldabayan@kfu.edu.sa

**Keywords:** chronic obstructive pulmonary disease, nursing interns, nursing education, workforce preparedness, evidence-based practice, chronic disease management, Egypt

## Abstract

**Highlights:**

**What are the main findings?**

- Late-phase interns had higher COPD screening score ranks than early-phase interns, but the difference was small (r = 0.12) and the independently sampled groups were markedly unequal.
- The eight-item outcome should be interpreted as a preliminary COPD knowledge-and-perceptions screening measure, not as a validated measure of clinical competence.

**What are the implications of the main findings?**

- Respiratory education, evidence-based information literacy, and smoking cessation training remain reasonable educational priorities, but this cross-sectional study does not establish that these strategies improve competence or patient outcomes.
- Longitudinal studies using validated COPD competence measures and participant-level psychometric analyses are needed before learning gains or workforce effects can be inferred.

**Abstract:**

**Background/Objectives:** Effective chronic obstructive pulmonary disease (COPD) management requires nurses who can support education, treatment adherence, smoking cessation, symptom monitoring, and pulmonary rehabilitation. This study described COPD care preparedness among nursing interns, compared independent early- and late-phase groups, and characterized selected educational and social context variables. **Methods:** A cross-sectional comparative study was conducted at two Egyptian nursing institutions between October 2024 and February 2025. Independent and markedly unequal early-phase (n = 114) and late-phase (n = 687) samples were recruited (N = 801). COPD preparedness was operationalized using a preliminary eight-item knowledge-and-perceptions screening measure. Raw-score bands (<24, 24–31, and 32–40) were used only as descriptive analytic groupings. Mann–Whitney U testing compared phases, and the effect size was calculated as r = |Z|/sqrt(N). **Results:** The upper, middle, and lower raw-score bands contained 52.9%, 37.3%, and 9.7% of participants, respectively. Late-phase interns had higher mean ranks than early-phase interns (412.60 vs. 311.07; Z = 3.498; *p* < 0.001), with a small effect size (r = 0.12). Forty-three participants (5.4%) reported current or prior tobacco use; because current and former use were combined, this value is not a current-smoking prevalence estimate, and dependence scores from that mixed subgroup were not interpreted. Mass media was the primary reported health information source for 71.4%, and 85.4% reported moderate or high interest in respiratory continuing professional development. **Conclusions:** A small phase-associated difference was observed in a preliminary COPD screening score, but the cross-sectional design cannot establish within-person learning gains. The measure does not assess clinical competence or patient outcomes, and the findings should be generalized only to interns similar to those sampled from the two participating Egyptian institutions. Longitudinal and psychometric validation studies are required.

## 1. Introduction

Chronic obstructive pulmonary disease (COPD) is a heterogeneous chronic respiratory condition associated with persistent airflow limitation, recurrent symptoms, exacerbations, disability, and substantial healthcare use [1]. It remains a major global cause of mortality and disability, with disproportionate burden in populations exposed to tobacco smoke, air pollution, occupational hazards, ageing-related risk, and health system constraints [2,3,4,5]. The challenge is especially important in low- and middle-income countries, where diagnostic capacity, specialist access, continuity of care, and structured chronic disease programs may be constrained [3,5].

The clinical burden of COPD is not determined by airflow limitation alone. Preventable exacerbations, poor treatment adherence, inadequate inhaler technique, delayed recognition of deterioration, continued tobacco exposure, and limited access to pulmonary rehabilitation contribute to avoidable morbidity and healthcare utilization [6,7,8,9]. For this reason, contemporary COPD care requires coordinated long-term management that combines pharmacological treatment with patient education, self-management support, smoking cessation, vaccination, adherence optimization, symptom monitoring, rehabilitation, and timely escalation of care [7,8,9]. These activities place nurses in a strategically important position across hospital, primary-care, rehabilitation, and community settings.

Nurses commonly provide repeated patient and family education, reinforce inhaler and medication adherence, assess symptoms and risk behaviors, support smoking cessation, promote self-management, facilitate pulmonary rehabilitation participation, coordinate follow-up, and contribute to supportive and palliative care in advanced disease [10,11]. The quality of these activities depends on more than staffing numbers: it requires a workforce with adequate disease-specific knowledge, evidence-based information skills, professional motivation, and confidence in the practical components of chronic respiratory care. Patient experiences of COPD also include uncertainty, stigma, guilt, and the daily burden of breathlessness, emphasizing the need for knowledgeable and credible clinical support [12,13]. Workforce preparedness can therefore be viewed as an upstream component of chronic care quality, even when patient outcomes are not measured directly.

The nursing internship is a particularly important point at which this capacity can be strengthened. It is the transition between academic preparation and increasingly autonomous clinical practice, during which interns encounter real patients and begin to consolidate professional identity, clinical judgment, and disease management routines [14]. Benner’s novice-to-expert framework conceptualizes this transition as movement from rule-based performance toward greater contextual recognition and prioritization [15]. Kolb’s experiential learning theory similarly proposes that concrete experience, reflection, conceptual integration, and active experimentation support the development of durable practice knowledge [16]. Clinical rotations therefore create opportunities to connect classroom knowledge about COPD with real-world activities such as symptom assessment, patient education, smoking cessation conversations, adherence counselling, and rehabilitation referral [17,18].

However, evidence regarding COPD-specific preparedness among nursing interns remains limited. The existing literature has more often examined qualified nurses, general undergraduate learners, simulation-based competence, or broader patient safety and evidence-based practice outcomes [19,20]. The internship phase has received less disease-specific attention, particularly in LMIC settings where nursing capacity may be central to extending chronic respiratory services. In addition, awareness alone does not necessarily translate into better utilization or outcomes, underscoring the importance of understanding the context in which disease knowledge is developed and applied [21]. Careful description of COPD screening scores and selected educational and social context characteristics across internship phases can therefore provide a cautious baseline for future longitudinal, psychometric, and multivariable studies without implying causal determinants.

Accordingly, this study examined COPD preparedness as an upstream nursing education construct rather than as a direct measure of clinical performance. The objectives were to (1) compare COPD knowledge-and-perceptions screening scores between independently sampled nursing interns in early and late internship phases; (2) describe reported current or prior tobacco use; and (3) characterize educational and social context variables, including health information source, interest in respiratory continuing professional development (CPD), peer smoking influence, and family respiratory-disease history. The study did not assess patient outcomes, rehabilitation attendance, treatment adherence, observed clinical competence, or within-person learning trajectories.

## 2. Materials and Methods

### 2.1. Study Design

A cross-sectional comparative design was used to assess COPD knowledge and care preparedness in two independent groups of nursing interns at different stages of internship training [22,23]. Early- and late-phase interns were sampled independently; participants were not followed longitudinally or matched across time. Consequently, between-group differences are interpreted as cohort-level associations with internship phase rather than individual learning trajectories, and causal inference is not warranted.

### 2.2. Setting and Participants

The study was conducted at two nursing education institutions in Egypt: the Faculty of Nursing and the Technical Institute of Nursing. Both institutions operate structured internship programs with supervised rotations across acute and chronic care environments, including internal medicine, intensive care, emergency care, and respiratory care settings. These rotations provide recurring opportunities to encounter patients with COPD and other chronic respiratory diseases. Data were collected from October 2024 to February 2025 during the 2024–2025 internship cycle.

Eligible participants were nursing interns actively enrolled in an internship program at either participating institution, able to complete the Arabic-language survey, and willing to provide written informed consent. Interns who participated in the pilot study were excluded from the main analysis. Convenience sampling was conducted during scheduled clinical rotations and academic sessions. The sample comprised 801 interns: 114 (14.2%) in the early phase and 687 (85.8%) in the late phase. This marked phase imbalance reflects the convenience sample and is treated as a potential source of selection and cohort effects throughout interpretation.

### 2.3. Preliminary COPD Knowledge-and-Perceptions Screening Measure

COPD preparedness was operationalized using an eight-item structured instrument developed for this study to screen foundational knowledge and care-related perceptions. The domains included COPD epidemiological importance and severity; modifiable and non-modifiable risk factors, including smoking and air pollution; occurrence among never-smokers; preventive strategies such as influenza and pneumococcal vaccination; and the roles of pharmacological treatment and pulmonary rehabilitation. Item development was conceptually informed by the Bristol COPD Knowledge Questionnaire and the Disease Awareness in COPD Questionnaire [24,25,26]. A single total score was used pragmatically to summarize breadth across these related domains; the composite was not intended to establish a unidimensional, validated clinical competence construct.

Each item was rated on a five-point Likert scale from 1 (strongly disagree) to 5 (strongly agree), yielding a total score from 8 to 40; higher scores indicated greater endorsement of COPD knowledge and favorable care-related perceptions. For descriptive presentation only, raw scores were grouped into lower (<24), middle (24–31), and upper (32–40) bands. These bands are researcher-defined analytic groupings and are not externally validated cut-off points, percentage scores, or clinically meaningful readiness thresholds. Accordingly, the continuous/ordinal screening score is emphasized when comparing internship phases, and the bands should not be interpreted as evidence of clinical competence.

### 2.4. Tobacco Use Status and the FTND

Participants reported tobacco use status using a single item that combined current or prior tobacco use. The original protocol administered the six-item Fagerstrom Test for Nicotine Dependence (FTND) to participants endorsing that combined item [27,28]. Because the screening item did not distinguish current from former users, the FTND responses from the mixed subgroup cannot be interpreted as a valid estimate of current nicotine/cigarette dependence. The revised manuscript therefore reports only the combined current or prior tobacco use status descriptively and does not present or interpret FTND dependence categories.

### 2.5. Translation, Content Validation, and Pilot Testing

The survey was prepared in Arabic using forward–backward translation procedures consistent with recommended cross-cultural adaptation practice [29]. Two bilingual translators with nursing and public health backgrounds independently translated the English items into Arabic. Differences were resolved by consensus. A third bilingual translator, blinded to the source wording, back-translated the Arabic version, and the research team reviewed semantic and conceptual equivalence. Three experts in nursing education, respiratory care, and public health evaluated the items for relevance, clarity, and domain coverage, after which minor wording refinements were made.

A pilot study involving approximately 5% of the target sample (about 40 interns) assessed feasibility, comprehension, completion time, and internal consistency. Pilot participants were excluded from the main study. The eight-item screening instrument showed Cronbach’s alpha = 0.82 in the pilot sample [30,31]. This pilot estimate should not be interpreted as construct validation. Exploratory or confirmatory factor analysis, corrected item–total correlations, and item-level psychometric validation were not performed in the main N = 801 sample. Although such analyses would be informative, they were not prespecified and were not introduced post hoc because the study was not designed as a formal psychometric validation study. Dedicated validation work should evaluate factor structure, item–total relationships, and other measurement properties before the instrument is treated as a validated scale [32].

### 2.6. Data Collection

Following ethics approval, trained research team members approached interns during scheduled clinical rotations and academic sessions. A standardized briefing explained the study purpose, the voluntary nature of participation, right to withdraw, confidentiality safeguards, and the absence of academic consequences for participation or refusal. Written informed consent was obtained before survey completion. Questionnaires were self-administered anonymously in supervised classrooms or clinical conference rooms. Research assistants answered procedural questions without coaching responses. Typical completion time was 10–15 min. Completed paper questionnaires were secured, and double-entry procedures were used to reduce data entry error.

### 2.7. Study Variables and Chronic Care Interpretation

The principal outcome was the total score from the preliminary eight-item COPD knowledge-and-perceptions screening measure; lower, middle, and upper raw-score bands were used only for descriptive presentation. The main comparison variable was internship phase (early versus late). Age, sex, institution, region of origin, prior healthcare experience, peer influence on smoking, family respiratory disease history, primary health information source, and interest in respiratory CPD were retained as descriptive contextual variables. The study did not measure observed clinical competence, patient self-management, adherence, pulmonary rehabilitation participation, exacerbations, readmissions, or quality of life.

### 2.8. Statistical Analysis

Data were analyzed using IBM SPSS Statistics version 24.0 (IBM Corp., Armonk, NY, USA). Frequencies and percentages summarized participant characteristics, contextual variables, tobacco use status, and the descriptive raw-score bands. Distributional characteristics of the COPD screening score were assessed using the Shapiro–Wilk test [33]. Because the score was non-normally distributed and derived from ordinal items, early- and late-phase groups were compared using the Mann–Whitney U test [34]. Mean ranks, Z statistics, two-sided *p* values, and the effect size r = |Z|/sqrt(N) are reported. No multivariable regression is presented in the final analysis. During model review, the original coding of several categorical predictors was found not to provide the explicit indicator variables and reference categories required for transparent category-specific interpretation; rather than report a potentially mis-specified model, the final analysis was restricted to descriptive summaries and the internship phase comparison. Future multivariable work should prespecify category coding, report 95% confidence intervals and model fit information, and verify the proportional odds assumption when ordinal regression is used. No imputation was performed; complete-case handling was prespecified for missing observations [35], although the variables reported in Table 1 and the phase comparison contain complete counts (N = 801). Statistical significance was set at *p* ≤ 0.05.

### 2.9. Ethical Considerations

Ethical approval was obtained from the Research Ethics Committee, Faculty of Nursing, Minia University (Approval No. REC2024103), and the King Faisal University Research Ethics Committee (Reference No. KFU-REC-2024-SEP-ETHICS2479). Dual oversight reflected participant recruitment within Egyptian training institutions and institutional requirements associated with King Faisal University sponsorship. All procedures were conducted in accordance with the Declaration of Helsinki [36]. Participation was voluntary, written informed consent was obtained, surveys were anonymous, and no personally identifiable information was collected. Research data were stored in password-protected files accessible to the research team and are retained in accordance with institutional governance requirements.

### 2.10. Use of Generative Artificial Intelligence in Manuscript Preparation

OpenAI ChatGPT (GPT-5.6 Sol) was used during manuscript preparation to assist with restructuring, language generation/editing, and reframing the presentation of the existing study toward chronic disease management and workforce capacity relevance. The tool was not used to generate, alter, or analyze study data. All statistical results, participant characteristics, instrument information, and study procedures reported here derive from the original study materials. The author reviewed and edited all AI-assisted text and accepts full responsibility for the accuracy, integrity, and interpretation of the manuscript.

## 3. Results

### 3.1. Participant Characteristics

The analytical sample comprised 801 nursing interns. Participants were predominantly 23–27 years old (52.8%), with 46.1% aged 18–22 years and 1.1% aged 28 years or older. The sample was nearly balanced by sex (50.9% male; 49.1% female). The Faculty of Nursing contributed 60.2% of participants and the Technical Institute of Nursing 39.8%. Slightly more participants were from rural than urban areas (53.4% vs. 46.6%). Most participants (85.8%; n = 687) were sampled during the late internship phase, whereas 14.2% (n = 114) were sampled during the early phase. Additional demographic and contextual characteristics are summarized in Table 1.

### 3.2. Reported Tobacco Use

Forty-three participants (5.4%) reported current or prior tobacco use, whereas 758 (94.6%) did not report either. Because the screening item combined current and former use, 5.4% should not be interpreted as the prevalence of current smoking. For the same reason, FTND dependence categories from this mixed subgroup are not presented or interpreted in the revised analysis.

### 3.3. COPD Screening Score Bands

For descriptive presentation, 424 participants (52.9%) fell in the upper raw-score band (32–40), 299 (37.3%) in the middle band (24–31), and 78 (9.7%) in the lower band (<24). These researcher-defined bands summarize performance on the preliminary knowledge-and-perceptions screening measure only; they are not validated clinical cut-off points and should not be interpreted as low, moderate, or high clinical competence.

### 3.4. COPD Screening Score by Internship Phase

COPD screening scores differed between the independently sampled internship phase groups. Late-phase interns had a higher mean rank than early-phase interns (412.60 vs. 311.07; Z = 3.498; *p* < 0.001). The corresponding effect size was r = 0.12, indicating a small group difference. Because the two phase groups comprised different participants and were markedly unequal in size, this result represents a cross-sectional cohort-level association rather than within-person improvement over time (Table 2).

## 4. Discussion

### 4.1. Principal Findings and Relevance to Chronic Disease Management

This study provides a cross-sectional description of COPD preparedness among nursing interns using a preliminary knowledge-and-perceptions screening measure. Late-phase interns had higher score ranks than early-phase interns, but the effect size was small (r = 0.12), and the two groups were independently sampled and markedly unequal. A small subgroup (5.4%) reported current or prior tobacco use; because current and former use were combined, neither current-smoking prevalence nor current dependence can be inferred. The learning context was also notable descriptively: 71.4% reported mass media as their primary health information source, while 85.4% reported moderate or high interest in respiratory CPD. These contextual variables are interpreted as sample characteristics rather than as causal determinants of preparedness.

A clear conceptual distinction is necessary. First, the questionnaire captures item-level endorsements of foundational COPD knowledge and care-related perceptions. Second, the summed score is an operational screening indicator of preparedness as defined for this study. Third, observed clinical competence—for example, performing inhaler teaching or smoking cessation counseling—was not assessed. Fourth, no patient-level outcomes were measured. The findings therefore concern a preliminary workforce education screening construct and should not be extrapolated to actual clinical performance, patient adherence, pulmonary rehabilitation uptake, exacerbations, readmissions, or other outcomes.

### 4.2. Internship Phase and Cross-Sectional Score Differences

The higher mean rank among late-phase interns is compatible with, but does not demonstrate, a contribution of clinical exposure to COPD-related knowledge and perceptions. Experiential and problem-based learning can strengthen health education skills, self-directed learning, and clinical reasoning [37,38], and Benner’s and Kolb’s frameworks provide plausible educational explanations for how repeated clinical encounters may contextualize knowledge [15,16,39]. In the present study, however, those mechanisms were not measured and should be regarded as hypotheses rather than explanations established by the data.

The phase comparison is constrained by the design. Early- and late-phase interns were different participants, the achieved groups were markedly unequal (114 vs. 687), and convenience recruitment creates potential selection effects. Cohort composition, institutional mix, prior experiences, or other unmeasured characteristics could contribute to the observed difference. The small effect size further argues against describing the result as a substantial learning gain. A longitudinal follow-up study of the same interns through defined rotations, ideally with baseline adjustment and standardized exposure measurement, is required to estimate within-person development.

### 4.3. Tobacco Use Reporting and Smoking Cessation Education

The tobacco use result should be interpreted narrowly. The 5.4% figure combines participants reporting current or prior tobacco use and therefore cannot estimate current-smoking prevalence. In addition, the FTND is based on current smoking behaviors; administering it after a combined current/former use screen makes dependence scores from the pooled subgroup difficult to interpret [27,28]. For this reason, dependence categories have been removed from the revised results, abstract, and highlights rather than treated as evidence about nicotine dependence among Egyptian nursing interns.

Smoking prevention and cessation nevertheless remain relevant to nursing education because tobacco exposure is central to COPD prevention and nurses can contribute to cessation support [10,40]. Regional studies and social network research also show that tobacco behavior is shaped by peer and social environments [41,42,43,44,45]. The present study measured peer smoking influence descriptively but does not claim that it predicts preparedness. Any recommendation for cessation support or brief intervention training is therefore framed as a general educational and workforce health priority, not as proof of a deficit demonstrated by the FTND data.

### 4.4. Information Environment and Continuing Professional Development

The descriptive information source profile warrants attention without implying a causal association with preparedness. In this sample, 71.4% identified mass media as their primary health information source, whereas 9.2% identified academic institutions, 9.0% the internet, 7.5% healthcare professionals, and 2.9% family or social networks. These categories indicate where participants reported obtaining health information; they do not measure frequency of use, source quality, perceived reliability, or evidence appraisal skill. Consequently, they should not be interpreted as demonstrating that one source causes better or worse preparedness.

Evidence-based practice education can strengthen learners’ ability to search for, appraise, and apply clinical information [46,47,48]. In the present sample, 85.4% reported moderate or high interest in respiratory CPD, but interest is not equivalent to participation, learning, or competence. Structured respiratory modules, guideline navigation exercises, supervised case review, and feedback-linked learning may therefore be reasonable educational options to test prospectively rather than interventions whose effectiveness is established by this cross-sectional study.

### 4.5. Contextual Variables and Future Analytic Work

Family respiratory disease history, peer smoking influence, primary health information source, and CPD interest were collected as contextual characteristics. These variables are described but are not treated as independent determinants of preparedness because the earlier multivariable model was not retained after review of its categorical variable coding. In particular, nominal variables such as primary health information source require category-specific indicator coding with an explicit reference category, while ordinal variables require a clearly justified coding strategy. Future participant-level analyses should prespecify those choices, report category-specific effect estimates with 95% confidence intervals, assess model fit and the proportional odds assumption when ordinal regression is used, and avoid interpreting family history or information source mechanistically unless the relevant pathways are directly measured.

### 4.6. Implications for Evidence-Based COPD Self-Management, Adherence, and Rehabilitation Support

This study does not demonstrate that interns can or cannot perform specific COPD clinical tasks. Nevertheless, COPD curricula generally need to connect foundational disease knowledge with the clinical functions that nurses may be expected to support, such as recognizing deterioration, reinforcing inhaler technique and medication adherence, explaining vaccination, addressing tobacco use, supporting self-management, facilitating pulmonary rehabilitation referral, and coordinating follow-up [8,9,10,11]. These domains are presented as curriculum-relevant areas based on COPD care requirements, not as measured competence deficits in this sample.

Three educational priorities are reasonable hypotheses for prospective evaluation: introducing respiratory content early and revisiting it across rotations; strengthening evidence-based information literacy; and providing accessible smoking cessation education and respiratory CPD opportunities. Because the present design did not test educational interventions, these recommendations should be understood as proposed workforce development strategies rather than evidence of effectiveness. Their effects on observed clinical performance and patient outcomes require longitudinal or experimental evaluation.

### 4.7. Strengths and Limitations

This study has several strengths. It included a large sample of 801 nursing interns from two educational institutions, addressed an underexamined transition point between prelicensure education and independent nursing practice, and described COPD-related knowledge/perceptions alongside relevant educational and social context characteristics. The analysis also distinguishes a cross-sectional phase association from longitudinal learning and reports an effect size estimate rather than relying on statistical significance alone.

Several limitations are important. The cross-sectional comparative design precludes causal inference and cannot establish individual improvement across internship. The early- and late-phase groups were independently sampled and markedly unequal in size (114 vs. 687), and convenience sampling from two institutions limits representativeness and generalizability beyond interns similar to those recruited. A formal response rate could not be calculated from the recruitment information available for the present analysis because phase-specific numbers for those approached, those eligible, and declining participation were not recorded in the analytic materials. The eight-item COPD instrument is a preliminary screening measure: its pilot internal consistency estimate (Cronbach’s alpha = 0.82) was based on about 40 interns, and construct validity, factor structure, corrected item–total correlations, and item-level performance were not established in the main sample. Psychometric analyses were not prespecified and were not added post hoc; therefore, the instrument should remain explicitly preliminary until dedicated validation is completed. In addition, because the earlier multivariable model was not retained after review of categorical variable coding, adjusted associations between contextual characteristics and screening scores are not estimated in this report. The lower/middle/upper score bands are researcher-defined descriptive groupings rather than validated or clinically meaningful thresholds.

All substantive measures were self-reported, creating potential recall and social desirability bias for tobacco use, CPD interest, information source reporting, and care-related perceptions. The tobacco use item combined current and prior use, preventing estimation of current-smoking prevalence and valid interpretation of FTND dependence in the pooled subgroup. This study also did not measure observed nursing behaviors or patient outcomes, including inhaler teaching, adherence support, self-management, rehabilitation participation, exacerbations, readmissions, or quality of life. Future work should use longitudinal designs, validated multidomain COPD competence measures, probability or broader multisite sampling, transparent category-specific modeling, and direct links between workforce measures and clinical or patient-level outcomes.

## 5. Conclusions

Among 801 nursing interns recruited by convenience sampling from two Egyptian nursing institutions, the late-phase group had higher COPD knowledge-and-perceptions screening score ranks than the early-phase group, but the effect was small (r = 0.12) and the groups were independent and markedly unequal. The cross-sectional data therefore do not demonstrate that internship exposure caused individual improvement. The preliminary eight-item score should not be interpreted as a validated measure of clinical competence.

The findings support cautious consideration of respiratory education, evidence-based information literacy, smoking cessation education, and accessible respiratory CPD as topics for future workforce development studies. These strategies were not tested in the present study. Longitudinal research using validated competence measures should determine whether specific educational exposures produce within-person gains and whether any gains translate into better clinical performance or patient-centered COPD outcomes.

## Figures and Tables

**Table 1:** Demographic and contextual characteristics of study participants (N = 801).

Characteristic	Category	n	%
Age (years)	18–22	369	46.1
	23–27	423	52.8
	≥28	9	1.1
Sex	Male	408	50.9
	Female	393	49.1
Internship institution	Faculty of Nursing	482	60.2
	Technical Institute of Nursing	319	39.8
Region of origin	Rural	428	53.4
	Urban	373	46.6
Internship phase	Early phase	114	14.2
	Late phase	687	85.8
Prior healthcare experience	Yes	420	52.4
	No	381	47.6
Family history of respiratory disease	Yes	378	47.2
	No	423	52.8
Peer influence on smoking	None	229	28.6
	Moderate	367	45.8
	Strong	205	25.6
Primary health information source	Mass media (TV/radio/print)	572	71.4
	Academic institutions	74	9.2
	Internet	72	9.0
	Healthcare professionals	60	7.5
	Family and social network	23	2.9
Interest in respiratory CPD	High interest	279	34.8
	Moderate interest	405	50.6
	Low/no interest	117	14.6

CPD = continuing professional development.

**Table 2:** Mann–Whitney U comparison of COPD screening scores by internship phase (N = 801).

Internship Phase	n	Mean Rank	Effect Size
Early phase (beginning of internship)	114	311.07	r = 0.12
Late phase (end of internship)	687	412.60	

Overall Mann–Whitney comparison: Z = 3.498; *p* < 0.001; effect size r = |Z|/sqrt(N) = 0.12. Groups were independently sampled; the result does not represent longitudinal change.

## Data Availability

The de-identified data supporting the findings are available from the corresponding author on reasonable request, subject to applicable institutional data governance and ethics requirements. The dataset is not currently deposited in a public repository.

## Abbreviations

The following abbreviations are used in this manuscript:

Abbreviation	Definition
BCKQ	Bristol COPD Knowledge Questionnaire
COPD	Chronic obstructive pulmonary disease
CPD	Continuing professional development
DACQ	Disease Awareness in COPD Questionnaire
FTND	Fagerstrom Test for Nicotine Dependence (original nomenclature; later termed Fagerstrom Test for Cigarette Dependence)
LMICs	Low- and middle-income countries
